# Evaluation of Filtering Bleb Function after Trabeculectomy with Mitomycin C Using Biomicroscopy, Anterior Segment Optical Coherence Tomography and In Vivo Confocal Microscopy

**DOI:** 10.4274/tjo.29052

**Published:** 2015-08-05

**Authors:** Suzan Güven Yılmaz, Cumali Değirmenci, Melis Palamar, Ayşe Yağcı

**Affiliations:** 1 Ege University Faculty of Medicine, Department of Ophthalmology, İzmir, Turkey

**Keywords:** Bleb, biomicroscopy, anterior segment optical coherence tomography, in vivo confocal microscopy

## Abstract

**Objectives:**

To analyze and assess compatibility of trabeculectomy filtering bleb characteristics and appearances using biomicroscopy, anterior segment optical coherence tomography (AS-OCT) and in vivo confocal microscopy (IVCM).

**Materials and Methods:**

Twenty-eight eyes of 28 patients who underwent glaucoma filtering surgery with mitomycin C in our clinic between 2009 and 2013 were evaluated. Morphological appearances of the blebs on slit-lamp biomicroscopy were defined according to the Moorfields bleb classification system. For the internal tissue assessment of blebs, AS-OCT and IVCM were performed. Bleb biometric parameters such as length, height and bleb wall thickness were assessed by AS-OCT; conjunctival epithelial-stromal cyst, structural network of conjunctival stroma and vascularisation were examined with IVCM. The relation between biomicroscopic morphological staging and bleb characteristics detected on AS-OCT and IVCM were assessed.

**Results:**

The mean age of the 28 patients (16 male, 12 female) was 57.2±15.9 (19 to 79) years. The mean time elapsed between surgery and examination was 29.2±19.2 (6 to 68) months. According to biomicroscopic appearance, 17 (60.7%) blebs were functional (13 diffuse, 4 microcystic), whereas 11 (39.3%) blebs were non-functional (9 flat, 2 encapsulated). In the comparison of non-functional and functional blebs, functional blebs were found to be superior in terms of biometric parameters on AS-OCT assessment (p<0.05). Higher number of epithelial and stromal cysts and less vascularisation were detected by IVCM in functional blebs when compared with non-functional blebs (p<0.05).

**Conclusion:**

Biomicroscopic appearances and characteristics on AS-OCT and IVCM of filtration blebs are consistent with each other. Besides biomicroscopic examination, which is an easy and practical method for determining bleb morphology, cross-sectional images obtained by AS-OCT and IVCM provide objective data regarding internal structure and functional features of blebs.

## INTRODUCTION

Trabeculectomy was first described by Cairns in 1968 and continues to be the gold standard in the surgical treatment of glaucoma.^1^ The long-term surgical success of the procedure, characterized by persistent reduction of intraocular pressure (IOP), is closely related to the formation of filtration blebs via conjunctival bulging at the sclerotomy site.^2^ Subconjunctival scarring is the most frequent cause of failure in filtration surgery.^3^ Mitomycin C (MMC) is an antimetabolite commonly used to prevent bleb scarring.^4^

As the interpretation of slit-lamp examination results regarding bleb height and diameter and the presence of microcyst or vascularization lies with the clinician, the determination of bleb morphology and function is subjective.^5,6^ Furthermore, the morphological appearance of the bleb gives no information about its internal structure. Anterior segment optical coherence tomography (AS-OCT) and in vivo confocal microscopy (IVCM) are becoming more commonly used in addition to IOP measurement and slit-lamp examination for the objective evaluation of bleb function.^7,8,9,10,11,12^

In this study we assessed trabeculectomy bleb functionality by examining characteristics and appearance by slit-lamp biomicroscopy, AS-OCT and IVCM and analyzed the correlation between these assessment methods.

## MATERIALS AND METHODS

Twenty-eight eyes of 28 patients that were diagnosed with glaucoma and underwent trabeculectomy surgery with MMC (0.2 mg/ml applied for 4 minutes) performed by the same surgeon (HA) in our clinic between April 2009 and December 2013 were included in the evaluation. Patients with severe dry eye or other ocular surface pathology which would hinder assessment and affect the reliability of the measurements were not included in the study. Furthermore, patients who had undergone previous surgery other than phacoemulsification with intraocular lens implantation (PHACO IOL) which would affect the conjunctiva were excluded.

The detailed histories and anti-glaucomatous drops used were recorded for the patients included in the study 29.2±19.2 months (6-68 months) after trabeculectomy. Data were collected in accordance with the Helsinki Declaration. Before participation in the study, each patient provided written informed consent. Best corrected visual acuity (BCVA) was assessed with the Snellen chart, IOP was measured by Goldman aplanation tonometry, and slit-lamp examination of the anterior segment and posterior segment examination with 90 D lens were conducted for each patient. Following the ophthalmological examination, color photographs of the anterior segment were taken by the same ophthalmologist. Bleb morphological appearance was graded according to the Moorfields Bleb Grading System (Table 1).^9^

Following slit-lamp evaluation, 0.5% proparacaine hydrochloride (Alcaine®, Alcon, Fort Worth, Texas, USA) was instilled as topical anesthesia and IVCM was performed on the entire eye using an HRT II (Heidelberg Engineering GmbH, Heidelberg, Germany) with the Rostock cornea module (RCM) attachment. Patients were seated with chin on the chin rest and forehead against the head rest; a drop of high viscosity artificial tears (0.2% polyacrylic acid) was applied to the objective lens, which was brought toward the center of the bleb while the patient looked down. Confocal imaging plane was adjusted manually to the filtering bleb located approximately 2 mm distant from the limbus in the superior bulbar conjunctiva. Images captured with the HRT II/RCM were 400x400 μm and all images were recorded digitally. During microscopic evaluation of the blebs by IVCM, presence of microcysts in the conjunctival epithelium, subepithelial connective tissue density and the presence of stromal blood vessels were noted.

The mean number of intraepithelial microcysts was calculated. Bleb subepithelial connective tissue was evaluated according to its reflectivity as described in previous studies^8,9,10,11^: grade 0, loose; grade 1, mild connective tissue; grade 2, moderate connective tissue; grade 3, dense connective tissue. Vascular structures were also evaluated during the connective tissue assessment based on the number of blood vessels present: grade 1 for 0 to 1 blood vessel; grade 2 for 2 to 3 blood vessels; grade 3 for more than 3 blood vessels. The investigator who performed the IVCM examinations was blinded to clinical data, and all calculations were performed using the best three images obtained.

**Figure 1:**
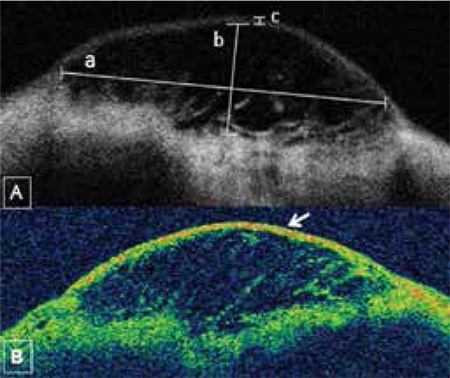
Biometric parameters used in the evaluation of blebs by anterior segment optical coherence tomography. A) a) bleb base width, b) maximal height of bleb interior, c) bleb wall thickness at thinnest point. B) Bleb wall density (arrow) determined by the device’s image colorization as follows: grade 1, black-blue low reflectivity; grade 2, blue-yellow moderate reflectivity; grade 3, red high reflectivity

Topcon 3D OCT-2000 (Topcon Corporation, Tokyo, Japan) was used to evaluate the anterior segment. Patients were seated and asked to place their chin and forehead on the rests. While looking down, the upper eyelid was gently lifted. After exposing the bleb and surrounding conjunctiva as much as possible, the device’s red light was aligned with the center of the bleb and longitudinal AS-OCT images were acquired by the same investigator for all patients. The following bleb biometric parameters were analyzed: i) bleb base width, ii) maximal height of the bleb interior and iii) bleb wall thickness at its thinnest point.^8^ Bleb wall density was also graded according to the device’s colorization of the images as follows: grade 1, black-blue low reflectivity; grade 2, blue-yellow moderate reflectivity; grade 3, red high reflectivity (Figure 1A, 1B).

Statistical analysis software was used in the comparison of bleb features as measured by biomicroscopic grading, IVCM and AS-OCT. Chi-square, Mann-Whitney U, Kruskal-Wallis and Student’s t-tests were used in comparisons.

## RESULTS

**Figure 2:**
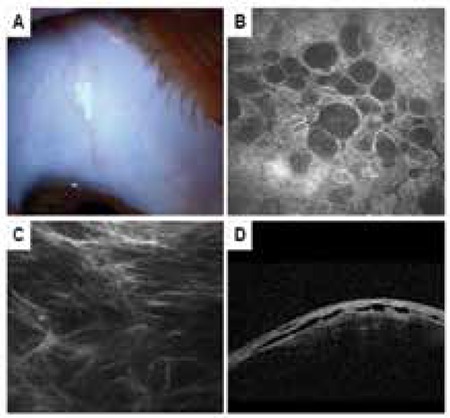
Diffuse bleb. A) Low, wide conjunctival elevation above the scleral bed viewed by biomicroscopy. B) In vivo confocal microscopy image showing many fluid-filled hyporeflective general microcysts. C) In vivo confocal microscopy image showing moderate (grade 2) loose subepithelial connective tissue. D) Anterior segment optic coherence tomography of a sagittal bleb cross-section showing fluid-filled, hyporeflective areas in the subconjunctival space

**Figure 3:**
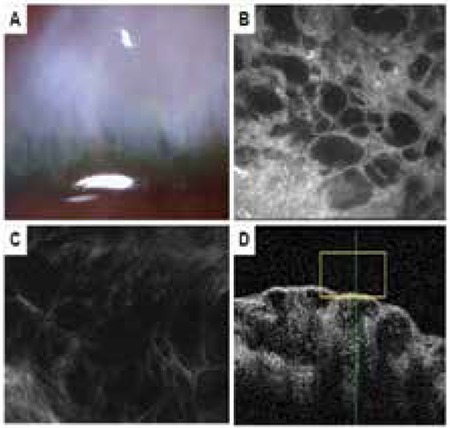
Microcystic bleb. A) Biomicroscopic view of a bleb with microcysts and pronounced elevation above the scleral bed. B) Many fluid-filled, hyporeflective microcysts in the conjunctival epithelium. C) In vivo confocal microscopy image showing loose (grade 0) subepithelial connective tissue. D) Anterior segment optic coherence tomography of the sagital cross-section of the bleb showing many fluid-filled subepithelial and subconjunctival spaces

**Figure 4:**
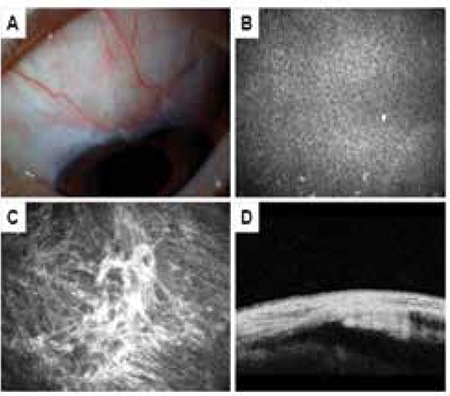
Flat bleb. A) The bleb does not show elevation in biomicroscopic examination. B) In vivo confocal microscopy showing inflammatory cell infiltration of the conjunctival epithelium and absence of microcysts. C) In vivo confocal microscopy showing dense (grade 3) subepithelial connective tissue. D) Anterior segment optic coherence tomography of bleb sagittal cross-section showing absence of subconjunctival fluid-filled space

**Figure 5:**
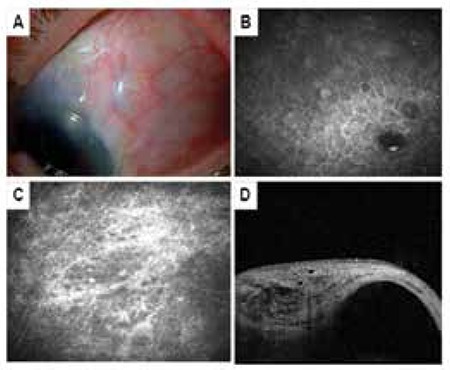
Encapsulated bleb. A) The bleb shows a demarcated area of elevation in biomicroscopy. B) Inflammatory cell infiltration in the conjunctival epithelium as well as 1-2 small microcysts with increased reflectivity visualized by in vivo confocal microscopy. C) Dense (grade 3) subepithelial connective tissue shown by in vivo confocal microscopy. D) Anterior segment optical coherence tomograph of a sagittal cross-section showing wide fluid-filled subconjunctival space surrounded by hyperreflective bleb walls

Demographic and clinical data of the patients are shown in Table 2. Four eyes (14.3%) had previously undergone PHACO IOL surgery; the other 24 eyes (85.7%) were phakic. According to the Moorfields Bleb Grading System of bleb biomicroscopic characteristics, 17 (60.7%) blebs (13 diffuse and 4 microcystic) were determined functional (Figure 2A and 3A); 11 (39.3%) blebs (9 flat and 2 encapsulated) were determined to be nonfunctional (Figure 4A and 5A). Eleven (64.7%) of the functional blebs were avascular and had thin walls. The mean IOP in the 17 patients with blebs that were biomicroscopically assessed as functional was 12.5±3.7 mmHg without the use of any anti-glaucomatous medication. In the 11 patients with nonfunctional blebs, the mean IOP was 23±6.8 mmHg; all used at least one type of anti-glaucomatous eye drop, with a mean of 2.5±1.5 agents used. The difference in IOP between the two groups was statistically significant (p<0.01; Student’s t-test). Three (75%) of the 4 pseudophakic eyes had functional blebs, while 1 (25%) had a flat bleb. The effects of age, gender, glaucoma type, and number and duration of anti-glaucomatous drugs used preoperatively on bleb function were assessed. Age, gender, glaucoma type and number of medications used did not affect bleb morphology (p=0.746, Mann-Whitney U test; p=0.06, chi-square test; p=0.405, chi-square test; and p=0.547, Mann-Whitney U test, respectively). However, the duration of medication use prior to trabeculectomy was significantly shorter in patients with functional blebs compared with those with nonfunctional blebs (4.5±3.9 months and 19±17.3 months, respectively; p=0.04, Mann-Whitney U test).

Analysis of IVCM parameters revealed that the mean number of microcysts in functional blebs was 9.5±8.7 (Figure 2B and 3B), while there were significantly fewer microcysts (2.4±5.2) in nonfunctional blebs (Figure 4B and 5B, p=0.001, Mann-Whitney U test). Functional blebs also showed significantly lower connective tissue density compared with nonfunctional blebs (Figure 2C, 3C, 4C and 5C, p<0.01, Mann-Whitney U test). In the assessment of stromal vascular structures, the functional bleb group had significantly lower grades than the nonfunctional group (p=0.001, Mann-Whitney U test, Table 3).

**Figure 6:**
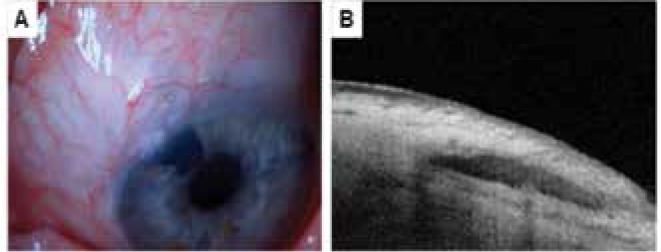
A bleb classified as flat by biomicroscopic examination that exhibited encapsulation in anterior segment optical coherence tomography. A) Bleb with indistinct appearance in biomicroscopy. B) Anterior segment optical coherence tomography showing a thick-walled subconjunctival fluid-filled space indicative of encapsulation

The base widths and heights of functional blebs were significantly larger than those of nonfunctional blebs (p=0.001 and p=0.019, respectively, Mann-Whitney U test; Figure 2D, 3D, 4D and 5D). The walls of functional blebs were significantly thicker than nonfunctional bleb walls (0.4±0.2 mm versus 0.2±0.1 mm, p=0.001, Student’s t-test), although no statistically significant difference between the two groups was detected in wall reflectivity (p=0.122, Mann-Whitney U test; Table 4). AS-OCT revealed thick-walled, fluid-filled subconjunctival spaces suggesting encapsulation in 2 (28.6%) of the 7 blebs that were biomicroscopically graded as flat (Figure 6A, 6B).

We evaluated whether there were any differences between glaucoma subtypes in terms functional bleb characteristics determined by IVCM and AS-OCT, but no statistically significant differences were detected in cyst number, mean blood vessel number, connective tissue density, base width, height and wall thickness (p=0.073, p=0.343, p=0.164, p=0.736, p=0.699, and p=0.966, respectively, Kruskal-Wallis test).

## DISCUSSION

In the evaluation of trabeculectomy functionality, biomicroscopic examination of the bleb and surrounding conjunctival tissue and IOP measurement continue to be the most commonly used methods.^13^ A diffuse appearance, slight elevation above the scleral flap, the presence of microcysts in the conjunctival epithelium and low conjunctival vascularization indicate bleb functionality, while a narrow surface area, excessive elevation and dense vascularization are indicators of bleb failure.^14^ Many bleb grading systems incorporating these parameters have been introduced and have been reported to show good correlation to bleb functionality.^9,14,15^ The Moorfields Bleb Grading System was used in this study; in eyes with functional blebs according to this system, IOP values were within normal range (12.5±3.7 mmHg), while eyes with nonfunctional blebs showed expected elevation in IOP (23±6.8 mmHg).

However, errors may arise during biomicroscopic evaluation because this method does not allow visualization of blebs’ interior structure and interpretation is affected by the clinician’s knowledge and experience. Therefore, for a more detailed and objective evaluation at the ultrastructural level, IVCM and AS-OCT have been increasingly used in recent years to analyze bleb architecture.^11,12,16^

IVCM produces real-time, high resolution images at the cellular level. It is a noninvasive method that allows the quantitative, qualitative and morphometric analysis of living tissue.^17^ In studies with blebs of varying morphology, IVCM has been used to identify microstructural changes such as epithelial and stromal cysts, encapsulation, and cellular infiltration.^10,11,12^ Labbe11 and Guthoff12 first used IVCM to evaluate blebs and found that blebs which appeared functional by biomicroscopy had significantly more epithelial microcysts and lower connective tissue density compared to apparently nonfunctional blebs. Similarly, we observed that blebs which appeared functional had a mean 9.5±8.7 cysts and low connective tissue density (0.8±0.7), while nonfunctional blebs showed significantly fewer microcysts (2.4±5.2) and denser connective tissue (2.2±0.9) by IVCM (p=0.001 and p<0.01, respectively, Mann-Whitney U test). Sbeity et al.16 also reported a lack of epithelial microcysts in nonfunctional blebs. Morita et al.18 found higher microcyst number and lower connective tissue density in functional blebs, as well as fewer blood vessels. Consistent with these findings, we found a significantly higher vascularization level in nonfunctional blebs compared to functional blebs (2.5±0.9 and 0.7±0.9, respectively; p<0.05, Mann-Whitney U test).

Although IVCM provides valuable data for the evaluation of bleb ultrastructure, because the procedure involves contact between the instrument and the eye, it can be affected by patient compliance, and there is the risk of infection with thin-walled blebs. Sbeity et al.16 conducted a study using a noncontact in vivo laser ophthalmoscope to evaluate filtering blebs and achieved results that corresponded to clinical morphology and were consistent with contact IVCM.

AS-OCT is used to capture high resolution cross-sectional images of the anterior segment tissues. As a digital technique, quantitative measurements can be obtained from the tomograms and automatic processing techniques can be used in the interpretation of the images. The technique does not involve contact with the eye and has a resolution of 10-20 microns.19 AS-OCT is being increasingly used to visualize pathologies of the anterior segment structures and surgical anatomy, and also allows anterior chamber biometry, corneal pacimetry and anterior chamber angle evaluation.^20^

AS-OCT cross-sectional images were used in this study to evaluate bleb biometric parameters such as wall thickness, radial length and height, as well as bleb wall reflectivity. All biometric parameters with the exception of bleb wall reflectivity showed statistically significant differences between functional and nonfunctional blebs (p<0.05). The functional blebs in our study exhibited significantly greater base width, height and wall thickness compared with nonfunctional blebs (p<0.05). These results are consistent with those of Singh et al.^21^, but not with Ciancaglini et al.^8^ Singh et al.^21^ found significantly thicker walls in functional blebs versus nonfunctional blebs, and reported that this reflected aqueous humour drainage along the conjunctiva-episclera. Ciancaglini et al.^8^, however, found no differences between functional and nonfunctional blebs in any of the biometric parameters of the bleb wall except reflectivity. In the current study, as expected as an indicator of good subconjunctival drainage, all bleb parameters measured by AS-OCT except bleb wall reflectivity were found to be significantly higher.

AS-OCT evaluation revealed that 2 nonfunctional blebs that were biomicroscopically classified as flat were actually encapsulated. The inability to recognize these encapsulations with IVCM was attributed to their being deeply located.

In this study, 3 of the 4 eyes that underwent cataract surgery had functional blebs. We attributed the fact that cataract surgery did not impact bleb function in most cases to PHACO IOL surgery having no adverse effects on the conjunctiva. In addition, the significantly longer duration of medication use before trabeculectomy in eyes with nonfunctional blebs compared to those with functional blebs suggests that the preservatives found in medication have adverse effects on the conjunctiva.

We determined that 64.7% (11/17 eyes) of the functional blebs in this study were both avascular and thin-walled, which we attribute to the use of MMC.

This study demonstrated that biomicroscopic, AS-OCT and IVCM characterization of filtering blebs are consistent with one another. The determination of bleb morphology by slit-lamp examination is easy and practical, while the cross-sectional images obtained through IVCM and especially AS-OCT provide valuable, objective data regarding bleb internal structure and functional characteristics. Analysis with these devices will be especially important in the functional assessment of cases where IOP and bleb morphologic appearance do not correlate. However, despite not providing objective and detailed data about bleb internal structure, consistency between the Moorfields Bleb Grading System and IVCM and AS-OCT indicates that biomicroscopic grading of bleb functionality is adequate and reliable in clinics in which IVCM and AS-OCT are not available.

## Figures and Tables

**Table 1:**
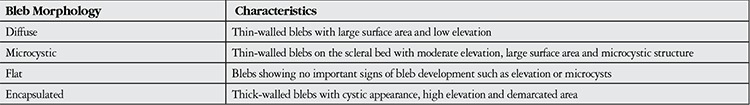
Moorfields Bleb Grading System^9^

**Table 2:**
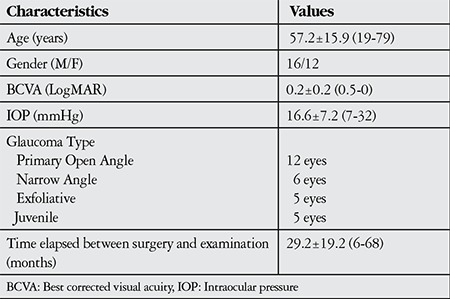
Patients’ demographic and clinical characteristics

**Table 3:**
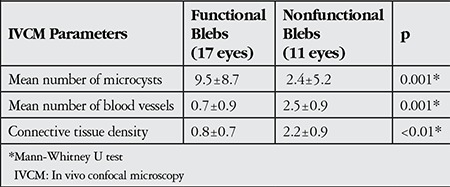
Comparison of in vivo confocal microscopy parameters in functional and nonfunctional blebs

**Table 4:**
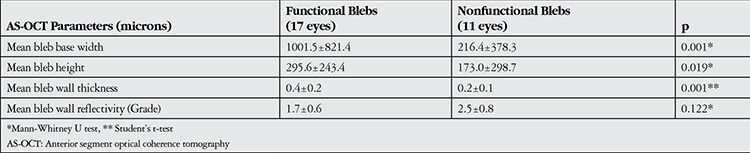
Comparison of anterior segment optical coherence tomography parameters in functional and nonfunctional blebs
